# Recurrent boosting effects of short inactivity delays on performance: an ERPs study

**DOI:** 10.1186/1756-0500-2-170

**Published:** 2009-08-26

**Authors:** Remy Schmitz, Manuel Schabus, Fabien Perrin, André Luxen, Pierre Maquet, Philippe Peigneux

**Affiliations:** 1 UR2NF [Neuropsychology and Functional Neuroimaging Research Unit], Université Libre de Bruxelles, B-1050 Bruxelles, Belgium; 2 Cyclotron Research Center, University of Liège, B-4000 Liège, Belgium; 3 Laboratoire de Neurosciences Sensorielles, Comportement, Cognition, UMR5020, Université Claude Bernard Lyon 1-CNRS, 69366 Lyon CEDEX 07, France; 4 Division of Physiological Psychology, Department of Psychology, University of Salzburg, Hellbrunnerstr. 34, 5010 Salzburg, Austria

## Abstract

**Background:**

Recent studies investigating off-line processes of consolidation in motor learning have demonstrated a sudden, short-lived improvement in performance after 5–30 minutes of post-training inactivity. Here, we investigated further this behavioral boost in the context of the probabilistic serial reaction time task, a paradigm of implicit sequence learning. We looked both at the electrophysiological correlates of the boost effect and whether this phenomenon occurs at the initial training session only.

**Findings:**

Reaction times consistently improved after a 30-minute break within two sessions spaced four days apart, revealing the reproducibility of the boost effect. Importantly, this improvement was unrelated to the acquisition of the sequential regularities in the material. At both sessions, event-related potentials (ERPs) analyses disclosed a boost-associated increased amplitude of a first negative component, and shorter latencies for a second positive component.

**Conclusion:**

Behavioral and ERP data suggest increased processing fluency after short delays, which may support transitory improvements in attentional and/or motor performance and participate in the final setting up of the neural networks involved in the acquisition of novel skills.

## Findings

Skill acquisition is a time-dependent process that can be split in two partially independent stages [1-3]. The first, fast learning phase is characterized by a rapid and nearly asymptotic improvement of performance. Fast learning, which mostly occurs during the initial practice session, is followed by periods of gradual acquisition and consolidation at a much slower rate, which takes place over repeated sessions spaced in time. Performance improvement over sessions may be observed even in the absence of intervening practice, disclosing off-line processes of memory consolidation for recently acquired information. In this context, consolidation is defined as the set of processes whereby memory traces become more stable and resistant to interference with the passage of time [3,4]. Neuroimaging, neurophysiological and behavioral studies in man and animal have additionally demonstrated that off-line consolidation processes already take place during the first hours of post-training wakefulness and continue later on during sleep [5-8].

Two studies recently added information about the off-line dynamics of performance evolution in isolating a transitory boost in motor performance after 5–30 minutes of post-training inactivity [9,10]. In agreement with prior studies [11-13] such gain in performance was not detectable any more 4–5 hours later within the same day. Additionally, boost amplitude was found to be a predictor of performance improvement 48 hours after initial practice [10], suggesting its involvement in the processing of motor memory traces. Also, although repetitive transcranial magnetic stimulation (rTMS) applied on the primary motor area (M1) during post-training inactivity markedly decreases the boost effect, it did not affect sleep-related improvement in performance 48 hours later [14]. This suggests that M1 activity participates in the expression of performance during the boost phase, but is not mandatory for the processes subtending long-term consolidation and delayed gains in performance. Similar transient enhancements in performance after practice on a pursuit rotor task have been already described by Eysenck and Frith [15] who coined this phenomenon under the term "reminiscence".

It remains unknown whether the boost effect is exhausted after the end of the initial, fast learning practice session, or may still happen during subsequent sessions, after that slow time-dependent consolidation processes have taken place. Also, it remains to be fully ascertained whether this process is merely motor in nature or also contributes to the consolidation of higher-order cognitive skills. We have tested these effects using the probabilistic serial reaction time (SRT) task, a paradigm of implicit sequence learning [16-18]. In the probabilistic SRT task, participants are confronted to visual stimuli appearing at specific locations on a computer screen; they must press as fast and as accurately as possible on the spatially corresponding key. Unknown to them, the sequence of stimuli is governed by a set of rules that describes permissible transitions between successive stimuli (i.e. an artificial grammar). Typically in this task, participants confronted to structured material (grammatical stimuli, G) have faster reaction times (RTs) than for random material (non grammatical stimuli, NG), suggesting response preparation towards the most predictable stimuli, thus learning of the sequential contingencies. When participants are subsequently asked to generate a sequence following the grammatical rules, they usually fail to exhibit any explicit knowledge of these rules, indicating implicit learning. Hence this task allows the assessment of both the evolution of motor performance through practice, and of the gradual, implicit acquisition of the sequential regularities embedded in an artificial grammar.

Although the neurophysiological underpinnings of the boost effect remain unknown, the electrophysiological correlates of the acquisition of sequential knowledge in a SRT task have been investigated in several studies. Using the event-related potentials (ERPs) technique, Baldwin and Kutas [19] showed a delayed onset of the P300 component for NG as compared to G stimuli, interpreted as reflecting detection of the grammatical deviance. As well, in a deterministic SRT (i.e. in which the length of the sequence and the number of trials are fixed), stimulus ungrammaticality enhanced amplitude of the N200 and P300 components [20-24]. This modulation was interpreted as the detection of the deviance of NG stimuli (N200) and the updating of the sequential model in memory (P300).

In this context, the aim of the present study was to investigate the reproducibility of the boost effect and its electrophysiological correlates in the context of a high-order, complex sequential learning paradigm, i.e. using the probabilistic SRT task [16].

**Figure 1:**
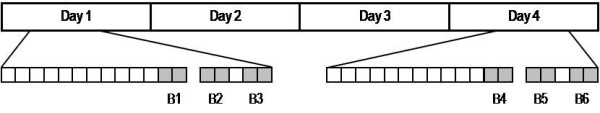
**Experimental design**. Practice sessions are scheduled at Day 1 and Day 4. **Squares**: blocks of practice session; **grey squares**: blocks of interest for the statistical analyses (performance is computed over 2 successive blocks). **Day 1. B1**: end of session 1 (blocks 12–13); **B2**: starting session 2 (blocks 14–15); **B3**: end of session 2 (blocks 17–18); **Day 4. B4**: end of session 3 (blocks 30–31); **B5**: starting session 4 (blocks 32–33); **B6**: end of session 4 (blocks 35–36).

Twenty-two participants (11 females, mean group age: 23.6 ± 3.26 years) participated in this study approved by the Ethical Committee of the Université de Liège (ULg). Participants faced a 17" computer screen where six permanent position markers were displayed horizontally above six spatially compatible response keys. A single SRT block consisted of 205 successive trials. On each trial, a black dot appeared 2 cm below one of the position markers, and the task consisted of pressing as fast and as accurately as possible on the corresponding key with the index, major and ring finger of the left and right hand. The interval between the response and the next stimulus was 250 ms. To assess learning of the probabilistic rules that govern succession of stimuli in the sequence, there was a 15% chance, on each trial, that the stimulus generated based on the grammar was replaced by a NG, random stimulus (see [17,18] for a detailed description of the probabilistic SRT task). Learning of the sequential contingencies was estimated in each block as the difference between reaction times (RTs) elicited by G and NG stimuli, in comparable temporal contexts defined by the previous stimulus. On day 1, all subjects performed 13 blocks of practice, then were tested after 30 minutes of inactivity for the presence (i.e., sudden increase in performance) and persistence (i.e. stabilization of the performance level) of the boost effect during 5 blocks. During this period of inactivity the participants seated quiet and listened to music under the control of an experimenter. Four days later (day 4), participants were tested in the same conditions than day 1 (see Figure 1). Therefore, within each day, three time windows were defined: (1) the moment just before the 30 minutes of inactivity (Pre-Boost; 12^th ^and 13^th ^blocks [B1 or B4]), (2) the moment just after the 30 minutes of inactivity (Boost; 14^th ^and 15^th ^blocks [B2 or B5]), and (3) the two last blocks of practice (Post-Boost; 17^th ^and 18^th ^blocks [B3 or B6]). At the end of B6 at day 4, each participant was asked to perform an explicit generation task. They were informed that the appearance of the stimuli on the screen followed a set of complex rules and that they had now to generate a succession of 405 trials of stimuli according to this hidden rule. The participant's performance on the generation task was compared to random computerized generation to assess the implicitness of learning [17,18].

**Figure 2:**
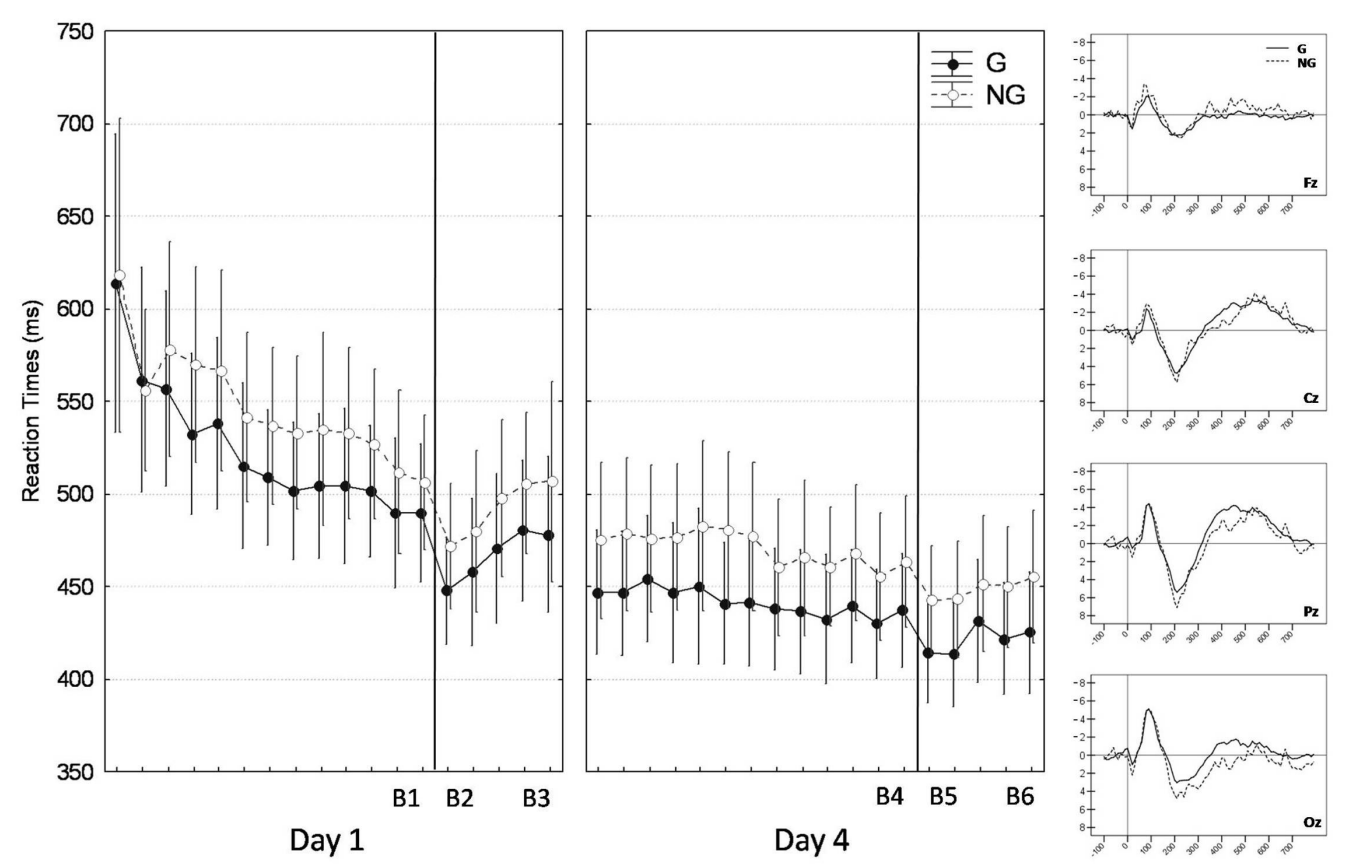
**Sequence learning effects**. **Left panel**: Average reaction times (and standard errors of the mean) per block for grammatical (G; filled circles) and non grammatical (NG; open circles) stimuli at Day 1 and Day 4. Errors bars are standard errors of the mean. Vertical bars represent the 30-minute break within the two learning sessions at Day 1 and Day 4. **Right panel**: Grammaticality effects. Average ERPs for grammatical (G; solid line) and non grammatical (NG; dashed line) stimuli at Fz, Cz, Pz and Oz electrodes.

A repeated measure ANOVA with Grammaticality (G vs. NG), Day (1 vs. 4) and Block Type (B1 vs. B2 vs. B3 vs. B4 vs. B5 vs. B6) within-subject factors on average reaction time values revealed a main effect of Grammaticality (F(1,19) = 43.48, *P *< .0001) (see Figure 2). Data inspection indicated that RTs were faster for G items than for NG items (451.715 ± 14.982 vs. 478.528 ± 16.506 ms), disclosing motor response preparation and anticipation of the next element in the sequence. Hence, subjects acquired knowledge about the sequential regularities in the material. Furthermore, participants' performance on the explicit generation task (i.e., number of G items generated: 268.05 ± 21.60) did not differ from random computerized generation (271.32 ± 7.63, *P *= .51), indicating that learning of the sequence was implicit [17,18].

**Figure 3:**
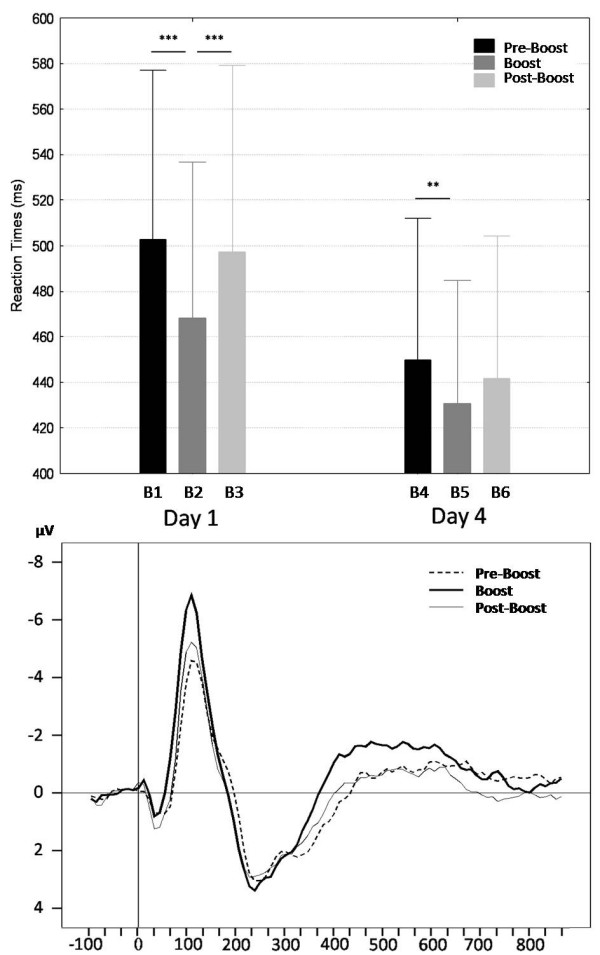
**Boost-related motor improvements**. **Upper panel**: Average reaction times for the two last blocks at the end of practice (Pre-Boost; B1 and B4), and the two first blocks (Boost; B2 and B5) and 4^th ^and 5^th ^blocks (Post-Boost; B3 and B6) after the 30-min interval, at Day 1 and Day 4. Tukey's Post Hoc: (***) *P *< .001, (**) *P *< .01. Errors bars are standard errors of the mean. **Lower panel**: Stimulus onset related potentials at Pre-Boost, Boost and Post-Boost sessions, averaging on Day1 and Day4 on electrode Oz.

The Day effect was also significant (F(1,19) = 68.55, *P *< .0001): irrespective of grammaticality, RTs were faster at day 4 than day 1 (440.836 ± 13.917 vs. 489.407 ± 17.668 ms), suggesting that motor performance had consolidated over days. Finally, the main effect of Block Type was significant (F(2,38) = 21.05, Tukey's HSD *P *< .0001), as well as a Day × Block Type interaction effect (F(2,38) = 4.61, *P *< .05; see Figure 2 and Table 1). Interactions with the Grammaticality factor were non significant (Tukey's HSD, all *Ps *> .05), indicating that between-sessions changes in performance are not primarily related to the learning of the sequential contingencies of the material. We therefore focus on the behavioral correlates of the boost effect independently of the grammatical content of the sequence (i.e. the global RTs, G and NG combined). This effect was assessed as the difference in average reaction times between the two last blocks of the 13-block practice session (B1 or B4; see Figure 1) and the two first blocks just after the 30 minutes of inactivity (B2 or B5). The persistence of performance improvement during this period was assessed comparing reaction time values over B2 (respectively B5) and B3 (respectively B6). Post-hoc comparisons (Tukey's HSD) revealed that the boost effect was significant at both days (*Ps *< .01; see Figure 3). At day 1, this boost effect quickly vanished as performance observed at the end of the 5 blocks returned to the level observed at the end of practice (B1; *P *= .84). At day 4, the observed increase in RTs from boost (B5) to subsequent blocks (B6) failed to reach significance (*P *= .16). These data indicate that the boost effect observed after the 30-minute inactivity delay is short-lived and wanes with continued practice, especially on day 1.

**Table 1:** SRT Performance

**SRT (Day × Session)**	**Pre-Boost**	**Boost**	**Post-Boost**
**Day 1**	502.718 (17.730)	468.144 (16.427)	497.357 (19.550)

**Day 4**	449.960 (14.846)	430.734 (12.898)	441.813 (14.909)

Note. Reaction times and standard error of the mean (SEM) are reported in milliseconds.

Additionally, individual measures of boost-related improvement at day 4 [i.e., (B4–B5)/B4] were correlated with boost-related improvement at day 1 [i.e., (B1–B2)/B1; *r *= .53, *P *< .05] and with improvement from day 1 to day 4 [i.e., (B3-Bday4)/B3 where Bday4 are the two first blocks of the first session at day 4; *r *= .60, *P *< .01]. These results are in line with the hypothesis that the boost effect reflects to some extent the individual's potential for motor improvement on the long term [10].

To highlight the electrophysiological correlates of implicit sequence learning over practice and test sessions, EEG was recorded at 1000 Hz using a Neuroscan Synamp system (NeuroSoft Inc., Sterling, VA). The EEG signal was recorded on Fz, Cz, Pz and Oz sites, referenced to the nose, according to the international 10–20 system. Blinks, vertical and horizontal eye movements were monitored via bipolar montages. Chin electrodes monitored muscular movements. EEG signal pre-processing included filtering (0.5–35 Hz) and rejecting visual- and muscle-related artifacts. Ocular-related artifacts in the EEG signal were corrected using Edit V4.2 (NeuroSoft Inc.) and an individual template-based canonical model of ocular movements. Data from two participants were rejected because of persistent artifacts on all electrodes. Analyses were conducted with respect to three stimulus-locked ERPs components (see Figure 2): a first positive component (0:100 ms) followed by a negative (100:200 ms) and a second positive (150:400 ms) components. The three peaks of amplitude found in the respective time windows were channel-wise. The latency of each component was defined with respect to the peak amplitude value within the defined time window.

For each component, we performed a repeated measures ANOVA with Grammaticality (G vs. NG), Day (1 vs. 4), Block Type (B1 vs. B2 vs. B3 vs. B4 vs. B5 vs. B6) and Electrode (Fz vs. Cz vs. Pz vs. Oz) on peak and latency values (see Table 2 for the main ERPs results). All results reported here are significant at *P *< .05 (Tukey's HSD). For amplitude but not latency, a main effect of Electrode was present for all the three components with weaker amplitude at Fz as compared to the other locations. A main effect of Grammaticality disclosed higher amplitude for NG stimuli for all components and longer latency for the second positive component (see Figure 2). In line with prior studies [25], the first positive component (P1) probably reflects an attentional mechanism inhibiting cerebral activity allocated to unexpected locations (NG stimuli) in order to avoid interferences and facilitates the integration of expected (G) stimuli. The larger amplitude of the negative component associated to NG stimuli may reflect the covert, automatic, detection of the grammatical deviance. Given its early latency we have labeled this negative component as a N100 [26-28]. Our interpretation differs from previous studies [20-24] because our negative component is not related to the consciousness of the sequence (which is here clearly implicit). The second positive component was interpreted as a P300 wave and thought to reflect the updating of the sequential model in memory [22]. As for the grammaticality effect on P300 latency, we interpret this response as a delay in the memory updating, at variance with Baldwin and Kutas [19] who viewed it as the detection of grammatical deviance. Indeed, grammaticality effects on P300 but not N100 latency suggest suggests functionally independent mechanisms.

**Table 2:** Main ERPs Results

**Electrode**	Fz	Cz	Pz	Oz
P1 amplitude	2.00 ± 0.30*	2.95 ± 0.45	2.95 ± 0.45	2.76 ± 0.41

N100 amplitude	-2.56 ± 0.41***	-3.19 ± 0.55***	-5.13 ± 0.64	-5.73 ± 0.59

P300 amplitude	3.71 ± 0.35*	6.36 ± 0.72	7.12 ± 0.72	5.26 ± 0.51*

				

**Grammaticality**		G	NG	*P*

P1	amplitude	2.27 ± 0.34	3.06 ± 0.43	< .0001

	latency	41.18 ± 2.62	39.75 ± 2.66	n.s.

N100	amplitude	-3.74 ± 0.53	-4.57 ± 0.51	< .01

	latency	116.43 ± 3.84	116.91 ± 3.29	n.s.

P300	amplitude	5.12 ± 0.49	6.11 ± 0.53	< .001

	latency	236.62 ± 6.02	245.72 ± 6.60	< .05

				

**Session × Electrode**		Pre-Boost	Boost	Post-Boost

N100 amplitude	Fz	-2.47 ± 0.46	-2.41 ± 0.45	-2.80 ± 0.41

	Cz	-3.04 ± 0.67	-3.05 ± 0.65	-3.49 ± 0.53

	Pz	-4.78 ± 0.85	-5.41 ± 0.65	-5.19 ± 0.67

	Oz	-5.29 ± 0.78	-6.47 ± 0.59**	-5.44 ± 0.63

				

**Day**		Day 1	Day 4	*P*

P1 amplitude		2.41 ± 0.42	2.92 ± 0.36	<.05

N100 amplitude		-3.71 ± 0.45	-4.6 ± 0.80	n.s.

P300 amplitude		5.32 ± 0.61	5.91 ± 0.53	n.s.

				

**Day × Electrode**		Day 1	Day 4	*P*

N100 amplitude	Fz	-2.58 ± 0.40	-2.54 ± 0.62	n.s.

	Cz	-2,76 ± 0.50	-3.62 ± 0.87	n.s.

	Pz	-4.39 ± 0.58	-5.86 ± 0.98	< .001

	Oz	-5.11 ± 0.55	-6.36 ± 0.90	< .01

Notes. Amplitudes are reported in microvolt (μV) and latencies in milliseconds (ms). All values are given with the standard error of the mean (SEM). Statistically significant tests are thresholded at * *P *< .05 or *** *P *< .001. n.s.: non significant.

Analyses on Block Type effect on P1 component failed to disclose any significant result, suggesting that this early attentional mechanism is not affected by the 30-minutes delay. A significant Block Type × Electrode interaction (F(6,114) = 5.63, *P *< .0001) was found for the N100 amplitude. Post-hoc analyses revealed a boost-related effect solely present on the electrode Oz (Figure 3). Boost in performance was associated with an enhancement of the N100 amplitude (Tukey's HSD, *P *< .001) which came back at baseline level at the end of the testing session (Boost vs. Post-Boost, *P *< .05). This suggests a short-lived effect of a 30 minute delay on the electrophysiological correlates associated to the automatic detection of the stimuli. There were a main effect of Block Type (F(2,38) = 4.75, *P *< .05) and a Block Type × Grammaticality interaction (F(2,38) = 4.29, *P *< .05) on P300 latency. Although P300 latency was longer for NG than G stimuli during practice (265.75 ± 8.73 ms vs. 247.25 ± 7.64 ms, Tukey's HSD, *P *< .01), latencies were not only shorter but also did not differ anymore between G and NG both during the boost (235.75 ± 8.58 ms vs. 226.68 ± 8.09 ms, *P *= .36) and subsequent blocks (235.68 ± 8.31 ms vs. 235.93 ± 8.35 ms, *P *= 1). Overall, these results suggest a global facilitation of the G and NG stimuli processing during the boost and immediately after.

To sum up, we have confirmed in this paper the presence of a transient boost effect in performance [9,10,14] in the framework of a complex sequence learning task. Importantly, we have shown that this effect is independent of implicit sequence knowledge. Also, it is reproducible but rapidly exhausted during the testing session, demonstrating the transient nature of the phenomenon. Moreover, individual boost-related gains of performance at day 4 are positively associated with both boost-related improvement at day 1 and improvement from day 1 to day 4. The reproducibility of the boost effect and its link with ulterior performance support the suggestion advanced by Hotermans and colleagues [10] that the boost might reflect a temporary "activated" state of motor memory.

The three ERP components (P1, N100 and P300) were modulated by the grammatical status of the stimuli. N100 amplitude and both P300 amplitude and latency were enhanced by the appearance of NG stimuli, consistent with previous studies [19,21,22]. These two components are likely to reflect the automatic, covert detection of the grammaticality deviance (N100) and the updating of the sequential model in memory (P300; [22]). A modulation of the early attentional mechanism P1 [25] was more surprising due to its very early nature (0:100 ms). This result suggests that the grammaticality of the stimuli is processed since the initial, early stage of cognitive processing. In line with our behavioral results, ERP data reveal a global and transient boost effect as revealed by the absence of a high order cognitive effect after a 30-minute delay. A transitory enhancement of the N100 amplitude was recorded at the Oz electrode as well as a reduction of the P300 latency during boost and the subsequent blocks of practice. This suggests a transitory improvement in cortical processing fluency after 30-minute delay of inactivity.

It may be argued that our psychophysiological data support an attentional, rather than a motor interpretation of the boost effect because the N100 component is modulated at the occipital location. In this perspective, the boost effect would occur mainly because subjects have had sufficient time to recover from fatigue and restore optimal alertness levels. However, previous studies have shown an absence of performance improvement after 4 h of inactivity [9,10,14], which does not fit this hypothesis. Indeed, would the boost be a genuine attentional effect, similar effects should be found after 30 minutes or 4 h of post-training inactivity. Additionally, Hotermans et al. [14] failed to show any alteration in boost-related performance after rTMS applied to the occipital cortex (i.e. their control condition), whereas stimulation of the motor cortex was highly effective. Although these data do not support the attentional component as the main basis of the boost effect, we do not reject the hypothesis of an additional attentional contribution in this specific time window during which performance is transitorily enhanced, 5 to 30 minutes of inactivity after the end of practice.

## Abbreviations

ERPs: event-related potentials; G and NG stimuli: grammatical and non grammatical stimuli; M1: primary motor area; rTMS: repetitive transcranial magnetic stimulation; SRT: serial reaction time

## Competing interests

The authors declare that they have no competing interests.

## Authors' contributions

RS performed the acquisition of behavioral and electrophysiological data, performed the statistical analysis and wrote the manuscript. PP, MS, FP and RS process the electrophysiological data. MS, FP, AL and PM wrote early draft of this manuscript. PP contributed to the conception and design of the study and the interpretation of data, and wrote the manuscript. All authors read and approved the final manuscript.
